# First Report of *Trichinella spiralis* in Free-Living Invasive American Mink (*Neovison vison*) in Lithuania

**DOI:** 10.3390/biology15090675

**Published:** 2026-04-25

**Authors:** Evelina Maziliauskaitė, Ramunė Tamošiūnaitė, Dalius Butkauskas, Petras Prakas

**Affiliations:** State Scientific Research Institute Nature Research Centre, Akademijos Str. 2, LT-08412 Vilnius, Lithuania; evelina.maziliauskaite@gamtc.lt (E.M.); ramune.tamosiunaite@gmail.com (R.T.); dalius.butkauskas@gamtc.lt (D.B.)

**Keywords:** American mink, *ITS1*, Lithuania, molecular identification, *Trichinella spiralis*

## Abstract

The genus *Trichinella* comprises zoonotic nematodes infecting a wide range of carnivorous and omnivorous animals, including humans. Infection occurs through the consumption of raw or undercooked meat containing viable *Trichinella* larvae. Among the species within this genus, *Trichinella spiralis* is considered one of the most epidemiologically important due to its high reproductive capacity and its frequent association with infections in domestic animals and humans. In this study, muscle samples from 18 invasive American minks (*Neovison vison*) were examined for *Trichinella* larvae using the magnetic stirrer method. Species identification was performed via multiplex polymerase chain reaction (PCR), while the internal transcribed spacer 1 (*ITS1*) region was amplified to evaluate the intraspecific genetic variability. *Trichinella* larvae were detected in one of the 18 (5.6%) animals investigated, and all isolates were identified as *T. spiralis*. Ten *ITS1* sequences obtained from individual larvae were 100% identical. Network and principal coordinate analyses revealed that the sequences clustered by geographic origin rather than host species and were more related to isolates from domestic pigs than to wildlife animals. These findings provide the first evidence of *T. spiralis* in American minks in Baltic and Scandinavian countries and contribute to a better understanding of the epidemiology of trichinellosis in the region.

## Simple Summary

Trichinellosis is a disease found worldwide that poses a significant risk to both animal and human health. It is caused by parasitic worms of the genus *Trichinella*, which can infect many different species. Despite efforts to control it, it remains a concern in many regions worldwide. Members of the Mustelidae family play an important role in the maintenance and transmission of this parasite. Among them, the invasive American mink may contribute to the spread of *Trichinella*, but investigations on this host remain limited. In this study, parasite larvae were detected in American mink using the artificial digestion method. Further analysis showed that the species detected is one of the most harmful to humans and animals. This is the first reported case of the parasite among wild American mink populations in the Baltic and Scandinavian regions. The results suggest that the genetic similarity among parasites is more closely linked to location than to the host species, and that wildlife helps maintain its diversity. Overall, further research is warranted to better understand the role of the American mink in spreading this parasite.

## 1. Introduction

Lithuania is inhabited by eight representatives of the family Mustelidae, including the invasive American mink (*Neovison vison* Schreber, 1777). Native to North America, this mesocarnivore was introduced to many countries worldwide in the early 20th century for fur farming purposes [1,2]. In Lithuania, the American mink was first introduced in the 1930s through fur farms established near Kaunas [3]. In Lithuania, the population increased from about 9000 individuals in 2007–2008 to approximately 12,000 in 2013–2015, and the species’ presence was recorded in around half of the surveyed water bodies [4]. By 2020–2021, the population size was estimated at 10,000 individuals, with up to 100 American minks hunted per year [5]. The American mink is an important reservoir of numerous pathogens, including helminths such as *Alaria mustelae*, *Filaroides martis*, *Isthmiophora inermis*, *Versteria rafei*, various acanthocephalans, *Echinococcus* spp., *Toxocara* spp., and *Trichinella* spp. [6,7]. Trichinellosis is a globally widespread zoonotic disease caused by nematodes of the genus *Trichinella* [8]. Thus far, ten species (*Trichinella britovi*, *Trichinella chanchalensis*, *Trichinella murrelli*, *Trichinella nativa*, *Trichinella nelsoni*, *Trichinella papuae*, *Trichinella patagoniensis*, *Trichinella pseudospiralis*, *Trichinella spiralis*, *Trichinella zimbabwensis*) and three genotypes (*Trichinella T6*, *T8*, *T9*) have been identified. Four species, *T. britovi*, *T. nativa*, *T. pseudospiralis*, and *T. spiralis*, are found in Europe [9,10]. These parasites infect a broad range of mammals, birds, and reptiles, with more than 150 animal species considered potential hosts. *Trichinella* spp. circulate within two distinct natural cycles: the sylvatic (wild) and the domestic [11]. Humans and domestic and wild animals most commonly acquire trichinellosis through the consumption of inadequately processed animal-derived products containing *Trichinella* spp. larvae. Unlike most parasites occurring in nature, the life cycle of all species of the genus *Trichinella* is completed within a single host and involves two successive parasite generations [12]. Among all species of the genus *Trichinella*, *T. spiralis* is considered the most pathogenic to humans. This species is most frequently associated with domestic pigs (*Sus scrofa domestica*), whose meat is widely consumed by humans [13,14]. During infection, adult female *T. spiralis* release the highest number of newborn larvae, resulting in a high parasitic burden. The larvae migrate and extensively encyst within striated muscles, causing severe muscle pain, and remain viable in muscle tissue for a longer period than larvae of other *Trichinella* species [15].

Invasive species exert significant negative effects on native fauna and flora by causing competition with local species, hybridization, and threats to ecosystem stability. In addition, invasive animals may introduce non-native pathogens, facilitate the emergence of new parasitic infections, and act as vectors or reservoirs of native parasites and pathogens [16]. Despite the widespread presence of the American mink in Lithuania, its role in the epidemiology of trichinellosis has not yet been investigated. This study aimed to assess the prevalence of *Trichinella* infection in invasive free-living American mink and to identify the detected species using molecular methods.

## 2. Materials and Methods

### 2.1. Sample Collection

**Figure 1:**
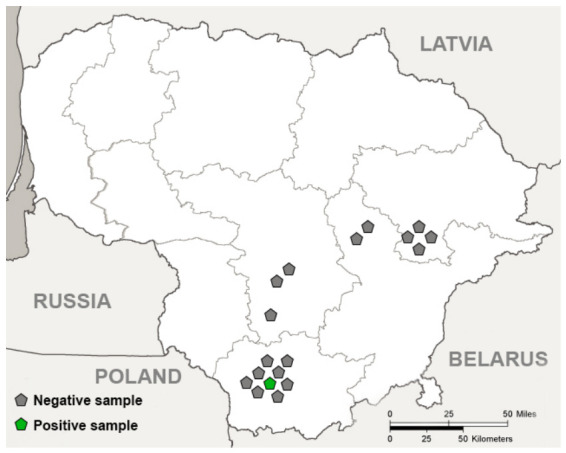
*Trichinella* spp. in the American mink (*Neovison vison*) in Lithuania. The grey pentagon shape represents negative individuals and the green pentagon shape represents one positive individual. Figure created using Adobe Photoshop (Adobe Inc., San Jose, CA, USA).

Eighteen American mink muscles from the hind legs and diaphragm were collected in 2021–2024 in cooperation with Kaunas Tadas Ivanauskas Zoology Museum (the Lithuanian national authority responsible for dead animals). Sampling sites were predominantly located in central and eastern Lithuania (Figure 1), and the collected animal samples were frozen at −20 °C until examinations had been conducted. Biological samples obtained from American minks were analyzed under the approval of the Ethics Committee of the State Research Institute Nature Research Centre (GGT-1; 11 January 2024).

### 2.2. Morphological Examination of Trichinella spp.

Muscle tissue samples (a mixture of hind leg and diaphragm tissue) of at least 40 g from each American mink were analyzed for the presence of *Trichinella* larvae using the modified magnetic stirrer method according to the procedure described previously [17]. This method is recognized by the European Food Safety Authority (EFSA) as the most effective procedure for detecting *Trichinella* spp. [18]. Muscle tissue samples were cut into small pieces. The digestive solution was then prepared by adding 4.3 mL of 25% hydrochloric acid and 4 g of pepsin to 1.2 L of tap water preheated to 46–48 °C in a 2 L glass beaker. Muscle tissue was ground and added to the acidic solution. The mixture was stirred with a magnetic stirrer at 44–48 °C for 30 min. After muscle digestion, infection intensity was estimated by counting larvae per gram (*lpg*) of muscle. Larvae were collected and stored in 96% ethanol for further analysis.

### 2.3. Molecular Examination of Trichinella spp.

From each infected animal, 10 *Trichinella* larvae were randomly selected for the establishment of possible mixed infection. The genomic DNA was extracted individually from each larva. The purification of DNA was performed according to the method of Pozio and La Rosa 2003 [19], with modifications described below. Each larva was washed in PBS and placed in 5 μL of PBS, followed by the addition of 2 μL of Tris-HCl (pH 7.6). The sample was then heated at 90 °C for 10 min and cooled on ice for 10–15 min. Subsequently, 9 μL of proteinase K solution (final concentration 100 μg/mL) was added. Samples were incubated at 48 °C for 3 h, after which the heating step at 90 °C for 10 min was repeated. Extracted DNA was stored at −20 °C until further use.

Multiplex polymerase chain reaction (PCR) was used for *Trichinella* species identification as previously described [17,19]. The primer pairs target the *ES5* (expansion segment 5) and *ITS1* (internal transcribed spacer 1) regions of the *Trichinella* genome, both of which are parts of ribosomal RNA (rRNA) (Table 1). Notably, in Europe, four *Trichinella* species are prevalent and they can be distinguished using two primer pairs displayed in Table 1 [19]. Reactions were carried out in a final volume of 25 µL, containing 12.5 µL of DreamTaq PCR Master Mix (Thermo Fisher Scientific, Vilnius, Lithuania), 0.5 µL 0.5 µM of each primer, 6.5 or 7.5 µL of nuclease-free water (for multiplex and conventional PCR, respectively), and 4 µL of template DNA. We used negative and positive controls in each run of PCR. DNA confirmed by Sanger sequencing as belonging to four *Trichinella* species (*T. britovi*, *T. nativa*, *T. pseudospiralis*, and *T. spiralis*) was used as positive control, while nuclease-free water instead of template DNA was applied as negative control. The thermal cycling programme consisted of an initial denaturation at 95 °C for 5 min, followed by 5 cycles of 94 °C for 45 s (denaturation), 58 °C for 45 s (annealing), and 68 °C for 1 min (extension), then 30 cycles of 94 °C for 45 s, 58 °C for 45 s, and 72 °C for 1 min, with a final extension at 72 °C for 5 min. *Trichinella* species were differentiated by the length of amplified products.

Conventional PCR was performed to assess the genetic diversity of the detected species within the *ITS1* region. For the amplification of *ITS1*, TriM1/TriM2 primer pair was designed using Primer3 Plus (version 3.3.0) software [20]. The chosen primer pair targeted a short fragment of the *18S* rRNA, complete *ITS1* and a portion of *5.8S* rRNA. The length of amplified product was about 900 bp. Thermal cycling included an initial denaturation at 95 °C for 2 min, followed by 40 cycles of 95 °C for 30 s (denaturation), 55 °C for 1 min (annealing), and 72 °C for 1 min (extension), followed by a final extension at 72 °C for 3 min.

The PCR products were separated by agarose gel electrophoresis. Multiplex PCR products were run on a 2% agarose gel for 50 min, while conventional PCR products were run on a 1% agarose gel for 25 min. Electrophoresis was conducted at 90 V in 1× TAE buffer. After electrophoresis, gels were visualized under UV light, and the sizes of the PCR products were compared to a DNA ladder to identify *Trichinella* species.

### 2.4. DNA Sequence Analysis

Sequencing was performed on positive samples that were generated using the TriM1/TriM2 primers. Five microliters of the amplified PCR products were treated with FastAP alkaline phosphatase and ExoI exonuclease (Thermo Fisher Scientific Baltics, Vilnius, Lithuania) to remove unincorporated primers and nucleotides. Sanger sequencing was carried out using the BigDye^®^ Terminator v3.1 Cycle Sequencing Kit (Thermo Fisher Scientific, Vilnius, Lithuania) and a 3500 Genetic Analyzer (Applied Biosystems, Foster City, CA, USA), according to the manufacturer’s instructions. A reference *ITS1* sequence obtained in the present work was submitted to NCBI GenBank under accession number PZ161099.

The obtained sequences were compared with reference sequences of various *Trichinella* spp. using the Nucleotide BLAST tool [21] available at the National Center for Biotechnology Information (NCBI) (https://blast.ncbi.nlm.nih.gov/Blast.cgi; accessed on 9 February 2026). In the present study, generated sequences were aligned with those retrieved from NCBI GenBank using the ClustalW multiple sequence alignment algorithm implemented in MEGA version 12.0.14. [22]. The phylogenetic network analysis based on *ITS1* sequences was conducted using the median-joining method [23] implemented in NETWORK 10.2.0.0 software (https://www.fluxus-engineering.com/sharenet.htm, accessed on 15 February 2026). Variable sites were identified using MEGA, and for the construction of the median-joining network, indels were treated as a fifth nucleotide state; however, a weight value twice as small was assigned to indels.

For the evaluation of genetic relatedness between the compared *Trichinella* samples, principal coordinates analysis (PCoA) based on Nei’s genetic distance [24] was conducted using GenAlEx v. 6.502 [25]. Genetic differentiation among *Trichinella* samples was assessed by calculating pairwise Φ_ST_ values in Arlequin v. 3.5.2.2 [26], applying the Tamura–Nei nucleotide substitution model. The significance of pairwise Φ_ST_ estimates was evaluated through 10,000 permutation tests, with statistical support determined at the 95% confidence level. Moreover, Tajima’s D was used to assess neutrality in DnaSP v. 6.12.03. [27].

## 3. Results

### 3.1. Prevalence, Infection Intensity and Identification of Trichinella Species

**Figure 2:**
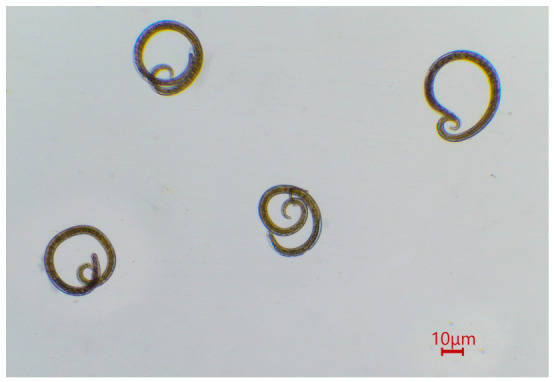
*Trichinella* spp. larvae recovered after muscle digestion with artificial gastric juice.

*Trichinella* larvae were detected in one of the 18 examined American minks (5.6%) collected in Lithuania (Figure 2). The infected animal was collected from the Žuvintas Biosphere Reserve. The infected individual harboured 38 larvae in 40 g of examined muscle tissue, corresponding to a mean intensity of 0.95 *lpg*.

**Figure 3:**
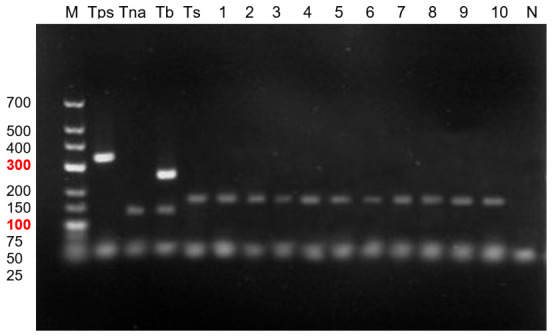
Agarose gel electrophoresis of multiplex PCR products for *Trichinella* species identification. Line M—GeneRuler Low-Range DNA Ladder (molecular weight marker), the 100 bp and 300 bp bands are highlighted in red as key reference markers for estimating fragment size. Positive controls: Tps—*Trichinella pseudospiralis* (340 bp), Tna—*Trichinella nativa* (127 bp), Tb—*Trichinella britovi* (127 and 253 bp), and Ts—*Trichinella spiralis* (173 bp). Samples analyzed in this study: lines 1–10—*T. spiralis*. N—negative control.

Ten *Trichinella* larvae were isolated from the infected animal to assess the presence of mixed infection. Each larva was subjected to multiplex PCR analysis. Electrophoresis analysis revealed that 100% of the samples examined were identified as *T. spiralis* (Figure 3).

After sequencing 10 *T. spiralis* larvae obtained from the American mink, no intraspecific genetic variability was detected. Comparison of the sequence generated in our study with those available in GenBank demonstrated 100% identity with *T. spiralis* isolates (MW302141, MW302143, MW302149, MW302157, MW302161, MW302168, MW302170, KC006423, XM_003370458) derived from domestic pigs in Poland, the USA, Finland, Spain, and Bulgaria, as well as from the American black bear (*Ursus americanus*) in the USA.

The obtained *ITS1* sequences of *T. spiralis* showed a high level of similarity to previously published *T. spiralis* sequences available in GenBank, with pairwise sequence similarity ranging from 98.1% to 100%. When they were compared with other representatives of the genus *Trichinella*, markedly lower similarity was observed, i.e., 84.9–85.5% with *T. murrelli*, 84.9–85.4% with *T. nativa*, and 83.5–84.8% with *Trichinella* sp. T6.

### 3.2. Genetic Relatedness of T. spiralis Samples Within ITS1

A total of 76 *ITS1* sequences of *T. spiralis* retrieved from GenBank were analyzed to assess the intraspecific genetic variability. These included 12 sequences from pigs in China (MW302127-38), two from pigs in South Korea (MW302139-40), one from pigs in Bulgaria (MW302141), two from pigs in Finland (MW302142-3), 17 from pigs in Poland (MW302144-60), three from pigs in Spain (MW302161-3), eight from pigs in the USA (MW302164-71), one from a pig in Egypt (MW302126), five from wild boar (*Sus scrofa*) in Israel (KU374871-3, KU374886-7), five from wild boar in China (MH289535-8, MH289540), 12 from American black bears (Ursidae) in the USA (KC006415, KC006422-7, KC006429-33), four from golden jackals (*Canis aureus*) (Canidae) in Israel (KU374868, KU374870, KU374880, KU374882), one from a red fox (*Vulpes vulpes*) (Canidae) in Israel (KU374876), one from Canidae sp. in China (OK644332), one from a masked palm civet (*Paguma larvata*) (Viverridae) in China (MH289539) and the sequence generated in the present study (PZ161099). After trimming sequences to start and end at the same nucleotide position, the length of alignment was 446 bp including gaps, while individual sequences’ lengths ranged from 429 to 438 bp.

**Figure 4:**
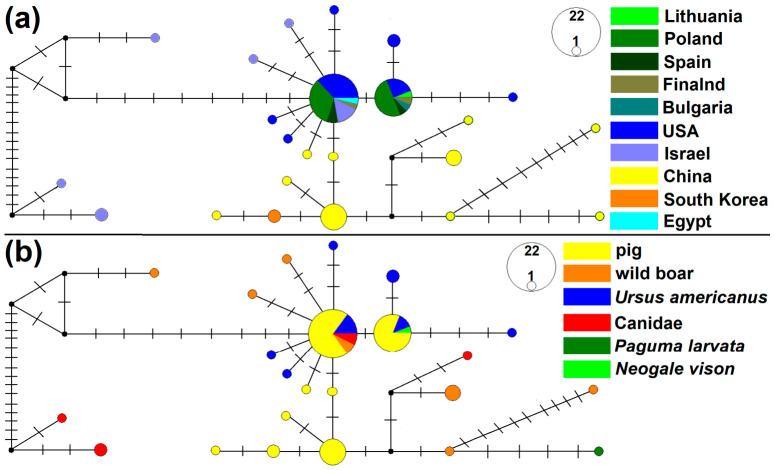
Median-joining networks inferred from *ITS1* sequences of *Trichinella spiralis*. Isolates were categorized according to country of origin (**a**) and hosts (**b**). Each dash corresponds to one mutational step. The size of each circle reflects genotype frequency. Black circles represent unsampled or extinct hypothetical intermediate genotypes.

The sequence established in American mink from Lithuania represented the second most common genotype with a frequency of 15. Meanwhile, the most frequent genotype (*n* = 22) differed from the Lithuanian genotype determined by a single mutational step, whereas the remaining genotypes were detected at low frequencies (one to five isolates). Median-joining network analysis revealed two central most common genotypes, which were predominantly shared by pigs from East Asia, Europe, and the USA, as well as by American black bears from the USA (Figure 4). Peripherally, most genetically distant genotypes were host- or region-specific, including those from wild boar and canids in Israel as well as those from wild boar, canids and from masked palm civet in China. Tajima’s D was significantly negative (D = −2.25884, *p* < 0.01).

**Figure 5:**
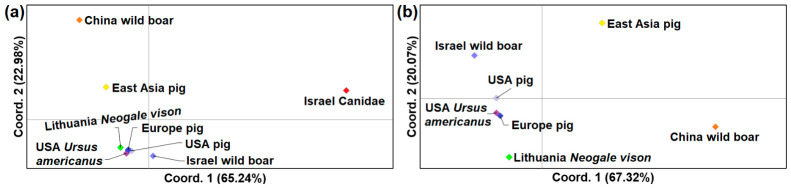
Principal coordinate analysis (PCoA) of *Trichinella spiralis* samples based on Nei’s genetic distances calculated from *ITS1* sequence data. Eight samples (**a**) or seven samples (**b**), excluding *T. spiralis* isolates obtained from canids in Israel, were included in the analysis.

For the intraspecific genetic comparison of *T. spiralis*, eight samples were compiled, comprising isolates from pigs in East Asia (*n* = 14), pigs in Europe (*n* = 23), pigs in the USA (*n* = 8), wild boar in China (*n* = 5), wild boar in Israel (*n* = 5), canids in Israel (*n* = 5), American black bears in the USA (*n* = 8), and American mink from Lithuania (*n* = 10, identical sequences). Principal coordinates analysis (PCoA) based on *ITS1* sequences showed that the sample from canids in Israel showed the most divergence from the remaining groups (Figure 5a). European and American pig isolates, together with those from American black bears, clustered closely and showed strong similarity to the Lithuanian mink and Israeli wild boar isolates (Figure 5b). In contrast, isolates from pigs and wild boars in East Asia were clearly separated from European and North American samples. The Φ_ST_ analysis was consistent with the PCoA results, demonstrating no genetic differentiation between the Lithuanian isolates and pig samples from Europe and the USA, as well as the American black bear sample (Table 2). Overall, pronounced genetic differentiation was observed among most pairs of *T. spiralis* samples, with the highest Φ_ST_ values detected between East Asian pigs and all other groups (Φ_ST_ = 0.315–0.790; *p* < 0.001).

## 4. Discussion

### 4.1. Prevalence and Infection Intensity of Trichinella spp. in American Mink

The present study is the first to report the occurrence of *Trichinella* spp. in free-living American mink in Baltic and Scandinavian countries, with a prevalence of 5.6% (1/18). In Europe, free-living American mink were tested for *Trichinella* spp. only in Poland, Latvia and Denmark (Table 3). The prevalence established in the current study was higher than that reported in Poland (3.3%; 27/812) and contrasts with the absence of infection in Latvia (0/5) [28,29]. However, these comparisons should be treated with caution given the considerable variation in sample sizes. Furthermore, *T*. *pseudosporalis* was recorded in free-living American mink from Denmark; nevertheless, no information is available regarding the number of animals examined or the methods used for species identification [11]. Notably, the prevalence observed in our study is markedly lower than that reported in farmed American mink in Estonia (14.3%; 5/35) [30], demonstrating that infection pressure may differ between wild and farmed populations. In addition, *Trichinella* infections in this host have been reported in Chile and China, with a prevalence of approximately 7% [31,32]. Currently, there is a lack of comprehensive studies worldwide assessing the role of the American mink in the transmission of *Trichinella* spp. infections. Nevertheless, the available literature indicates generally low prevalence levels; however, this species may still act as a potential reservoir and contribute to the circulation of these parasites in wildlife ecosystems.

In addition to the prevalence of *Trichinella* spp. in American mink, the intensity of infection in the current study (0.95 *lpg*) was considerably lower than that reported in Poland (mean 13.6 *lpg*; range 0.1–274.8 *lpg*) and Estonia (mean 4.8 *lpg*; range 0.5–9.0 *lpg*); was similar to values observed in Chile (mean 1.3 *lpg*; range 0.2–3.0 *lpg*); and was higher than that reported in China (range 0.025–0.815 *lpg*) [28,30,31,32]. The observed differences between countries should be interpreted with caution, as infection intensity may be influenced by ecological context, management conditions (farmed versus free-living), and methodological approaches. Given the relatively small sample size in the present study, broader investigations are required to more reliably assess the epidemiological role of American mink in the maintenance and transmission of *Trichinella* in Lithuania.

### 4.2. Identification and Phylogenetic Analysis of Trichinella spiralis

In the present study, *T. spiralis* was detected in free-living American mink, representing the first report of this species in the Baltic and Scandinavian regions. In Poland, investigations of free-living American mink revealed the presence of multiple *Trichinella* species—predominantly *T. britovi*, with individual detections of *T. spiralis* and *T. pseudospiralis*, including mixed infections—confirming that multiple *Trichinella* taxa can circulate in wild American mink populations in Europe [28]. Elsewhere in Europe, data on *Trichinella* in American mink are scarce; earlier records from Denmark report a single detection of *T. pseudospiralis* in American mink, but no detailed parasitological and molecular information is available to firmly characterize the finding [11]. Many studies in other European countries have either focused on different host species (e.g., wild carnivores) or have not analyzed *Trichinella* isolates to the species level, limiting comparisons across regions. Outside of Europe, *T. spiralis* has been molecularly confirmed in American mink in China, where farmed mink exhibited infection with *T. spiralis*, likely linked to exposure to synanthropic rats acting as reservoirs [32]. Similarly, in Chile, *T. spiralis* was identified in free-living American mink and associated rodent hosts; this was the first record of this species in a mustelid in South America [31]. Although *T. spiralis* and other *Trichinella* species have now been documented in American mink in multiple regions, overall data remain scarce. These findings indicate that while a range of *Trichinella* taxa can infect American mink, there are still relatively few studies exploring species-level diversity in this host, and additional research is needed to better understand geographic patterns and host associations.

*Trichinella spiralis* has been extensively investigated using a variety of genetic markers, including rRNA regions, mitochondrial DNA (mtDNA), and microsatellites. In addition, whole-genome sequencing of *T. spiralis* has been conducted to support future studies on population genetics, epidemiology, and parasite evolution [35]. Several studies have examined the genetic variability of *T. spiralis* and *T. britovi* across different geographic regions, revealing greater genetic diversity in *T. britovi* than in *T. spiralis* [36,37]. For instance, microsatellite analyses of *T. spiralis* have shown low levels of heterozygosity (mean ~0.139 per locus) [38]. Furthermore, mtDNA and *ITS1* loci revealed only a limited number of haplotypes or genotypes of *T. spiralis*, differing by only a few SNPs, indicating reduced genetic variability [39].

Higher genetic diversity of *T. spiralis* has been reported in Asian isolates compared to those from Europe and North America [40]. This has led to the hypothesis that *T. spiralis* originated in Asia, whereas Western populations are relatively homogeneous and may reflect a recent population bottleneck, potentially associated with the parasite’s spread through domesticated animals [36,40]. The results of the present study, including pairwise Φ_ST_ and PCoA, are consistent with these findings, confirming reduced genetic variability in European populations compared to Asian ones. Consequently, more detailed investigations into Western populations of *T. spiralis* require highly polymorphic markers and multilocus approaches.

Our results also indicate that genetic clustering is driven more strongly by geographic origin than by host species, further supporting the detection of regional population structure rather than strict host specificity. Moreover, we observed that isolates associated with domestic animals, particularly pigs, exhibit lower genetic diversity compared to those circulating in wildlife. This supports the widely accepted view that the genetic diversity and long-term maintenance of *T. spiralis* are primarily sustained within the sylvatic cycle in wildlife hosts, which plays a crucial role in parasite persistence and adaptability [36,37,38,40].

Despite its significance, studies on *T. spiralis* genetic diversity in wildlife remain limited. For example, a recent report described a rare infection of *T. spiralis* in a brown bear in Alaska, which is notable given that this parasite is typically associated with swine and is considered largely controlled in domestic cycles in North America [41]. Such findings highlight the ongoing circulation of *T. spiralis* in wildlife and the importance of these hosts as reservoirs.

Taken together, these findings emphasize the need for further research on *T. spiralis* in wildlife hosts, including mustelids and other predatory species, to improve our understanding of parasite transmission dynamics, maintenance in natural ecosystems, and potential risks to animal and public health.

### 4.3. Epidemiological Significance of Trichinella spiralis

*Trichinella spiralis* is considered the most pathogenic species within the genus, primarily because infected females produce a higher number of newborn larvae compared to other *Trichinella* species [42]. This high reproductive capacity results in a greater larval burden in host tissues, which is directly associated with more severe clinical manifestations. Moreover, *T. spiralis* is the principal etiological agent responsible for the majority of human trichinellosis cases and related fatalities worldwide. Its enhanced pathogenicity has been attributed not only to the greater number of larvae released by females but also to the stronger inflammatory and immune responses it induces in humans compared with other genotypes [43,44].

The species is widely distributed across diverse geographic regions. In Western Europe, *T. spiralis* has been reported in Austria, Germany, France, the Netherlands, Ireland, and the United Kingdom. In Southern Europe, it has been documented in Spain and Italy. In Central Europe, records are available from the Czech Republic, Hungary, Poland, the Slovak Republic, and Slovenia. In Eastern Europe, the parasite has been reported in Romania, Ukraine, and Russia. In Northern Europe, it has been documented in Finland, Sweden, Estonia, and Lithuania. In Southeastern Europe, records exist from Croatia, Serbia, Bosnia and Herzegovina, and Bulgaria [45,46]. Outside Europe, the presence of *T. spiralis* has also been confirmed in China and Chile, demonstrating its broad global distribution across different ecological zones [31,32].

Importantly, *T. spiralis* infects a remarkably wide range of host species, encompassing domestic, wild, and synanthropic animals [14]. It has been recorded in domestic pig, domestic cat (*Felis catus*), domestic dog (*Canis lupus familiaris*) domestic horse (*Equus caballus*), and humans, as well as in members of the family Canidae, including grey wolf (*Canis lupus*), red fox, raccoon dog (*Nyctereutes procyonoides*) and golden jackal; members of the family Felidae, including lynx (*Lynx lynx*) and wild cat (*Felis silvestris*); members of the family Mustelidae, such as European badger (*Meles meles*) and American mink; members of the family Ursidae, including brown bear (*Ursus arctos*) and American black bear; and Suidae, particularly wild boar. Synanthropic and wild rodents, with notable examples including the brown rat (*Rattus norvegicus*), black rat (*Rattus rattus*), and field mouse (*Apodemus sylvaticus*), also play a significant role in maintaining transmission [31,32,45,46].

Transmission dynamics are strongly influenced by human practices. Inadequate management of domestic pigs and improper handling of wildlife carcasses facilitate the spillover of *Trichinella* spp., especially *T. spiralis*, from sylvatic to domestic environments. High-risk farming practices, such as intentional feeding of pigs with food waste containing raw pork scraps, uncontrolled outdoor access, and accidental exposure to infected carcasses, are key factors for initiating and maintaining the domestic cycle [46].

Comparative data indicate that *T. spiralis* is more prevalent in domestic and synanthropic hosts than other species, such as *T. britovi*. For example, in sylvatic wild boar, *T. spiralis* accounts for 62% of isolates compared to 38% for *T. britovi*, whereas in domestic pigs the proportion increases to 82% versus 18%, respectively. A similar pattern has been observed in synanthropic rodents (75% *T. spiralis* vs. 25% *T. britovi*), particularly in brown rats, in which 82% of isolates correspond to *T. spiralis* and 18% to *T. britovi*. These findings suggest a stronger association of *T. spiralis* with the domestic and synanthropic transmission cycle [45].

The detection of *T. spiralis* in free-living American mink in the present study, therefore, raises important epidemiological considerations. Given the species’ strong link to domestic and synanthropic hosts, the presence of *T. spiralis* in a wild mustelid may reflect spillover from domestic or peridomestic environments into wildlife. Conversely, carnivorous species such as American mink could potentially contribute to maintaining or disseminating the parasite within natural ecosystems through predation and scavenging. Further investigations are necessary to clarify whether American mink act primarily as spillover hosts or play a more active role in sustaining transmission in the sylvatic cycle.

## 5. Conclusions

This study provides the first investigation of muscle samples from American minks that aimed to identify the presence of *Trichinella* spp. in Lithuania. *Trichinella* larvae were detected in one out of 18 examined muscle samples. Molecular analysis confirmed the parasite as *T. spiralis*, indicating that American mink can serve as potential carriers of this zoonotic parasite. *ITS1* sequence-based intraspecific genetic analysis indicated that *T. spiralis* genotypes clustered predominantly according to geographic origin rather than host species. The observed pattern supports the role of the wildlife host in maintaining the higher genetic diversity of this parasite. Notably, the *T. spiralis* genotype identified in this study showed greater similarity to those circulating in domestic pigs than to isolates reported from wildlife. These findings provide new insights into the population structure of *T. spiralis* and highlight the complex interactions between domestic and sylvatic transmission cycles. Overall, our study provides the first evidence of *T. spiralis* in the muscle tissue of American minks in the Baltic and Scandinavian region, highlighting the potential role of this invasive species in the epidemiology and transmission of *Trichinella* spp.

## Figures and Tables

**Table 1:** Primer pairs used for PCR amplification in this study.

PCR	Genetic Region	Primer Name	Primer Sequences	Ref.
Multiplex	*ES5*	Primer I	5′-GTTCCATGTGAACAGCAGT-3′	[19]
		Primer I	5′-CGAAAACATACGACAACTGC-3′	
	*ITS1*	Primer II	5′-GCTACATCCTTTTGATCTGTT-3′	
		Primer II	5′-AGACACAATATCAACCACAGTACA-3′	
Conventional	*ITS1*	TriM1	5′-AAATTTCCCAAACCAAATCA-3′	PS
		TriM2	5′-GTCTCGCCTCGTTTTTCATC-3′	

PS—present study.

**Table 2:** Genetic differentiation analysis among eight *Trichinella spiralis* samples based on *ITS1* sequences. Pairwise Φ_ST_ values are displayed below the diagonal, while statistical significance of Φ_ST_ values is shown above the diagonal. Statistically significant Φ_ST_ values (*p* < 0.05) are highlighted with a grey background.

	EAPig	EUpig	USUa	USpig	ILSs	CNSs	ILCa	LTNv
EAPig		<0.00001	<0.00001	<0.00001	0.00010	0.00010	0.00040	<0.00001
EUpig	0.790		0.00030	0.99990	0.00248	<0.00001	0.00446	0.99990
USUa	0.512	0.092		0.66380	0.04653	<0.00001	0.01228	0.48461
USpig	0.675	0.000	−0.015		0.03495	0.00109	0.03168	0.99990
ILSs	0.450	0.342	0.086	0.099		0.00861	0.09554	0.02089
CNSs	0.315	0.725	0.451	0.501	0.262		0.05485	0.00040
ILCa	0.684	0.777	0.607	0.576	0.382	0.422		0.02089
LTNv	0.698	0.000	0.008	0.000	0.146	0.552	0.622	

*Trichinella spiralis* was isolated from pigs in East Asia (EAPig), pigs in Europe (EUpig), American black bears in the USA (USUa), pigs in the USA (USpig), wild boar in Israel (ILSs), wild boar in China (CNSs), members of the family Canidae in Israel (ILCa) and American mink from Lithuania (LTNv).

**Table 3:** Global summary of published studies on *Trichinella* infection in American minks (*Neovison vison*).

Country	Free-Living or Farmed	N Tested	N Positive (%)	Range *lpg* (Mean *lpg*)	Identification Methods	*Trichinella* Species	Ref.
Poland	Free-living	812	27 (3.3)	0.1–274.8 (13.6)	Magnetic stirrer artificial digestion	*T. britovi*, *T. spiralis*, *T. pseudospiralis*	[28]
Lithuania	Free-living	18	1 (5.6)	0.95 (0.95)	Magnetic stirrer artificial digestion	*T. spiralis*	PS
Denmark	Free-living	No data *	No data *	No data *	No data *	*T. pseudospiralis*	[11]
Latvia	Free-living	5	0	0	Magnetic stirrer artificial digestion	Not identified	[29]
Estonia	Farmed	35	5 (14.3)	0.5–9.0 (4.8)	Magnetic stirrer artificial digestion	Not determined	[30]
Fennoscandian **	Farmed	2063	0	0	Artificial digestion	Not identified	[33]
China	Farmed	289	20 (6.9)	0.025–0.815 (-)	Pooled magnetic stirrer artificial digestion	*T. spiralis*	[32]
Chile	Free-living	100	7 (7.0)	0.2–3.0 (1.3)	Magnetic stirrer artificial digestion	*T. spiralis*	[31]
South America	Free-living	94	0	0	No data	Not identified	[34]

*—cited without an accessible primary research publication; no detailed study information available. **—refers to Norway (mainland), Sweden, and Finland. PS—present study.

## Data Availability

The *ITS1* sequence of *Trichinella spiralis* is available at NCBI GenBank under accession number PZ161099. Notably, an identical sequence was identified in 10 larvae isolated from the American mink (isolate: NvTri23.2).
